# Government responses to the COVID-19 pandemic of the Gulf Cooperation Council countries: good practices and lessons for future preparedness

**DOI:** 10.1186/s41256-024-00349-y

**Published:** 2024-03-15

**Authors:** Shu Chen, Lei Guo, Yewei Xie, Di Dong, Rana Saber, Mohammed Alluhidan, Adwa Alamri, Abdulrahman Alfaisal, Nahar Alazemi, Yahya M. Al-Farsi, Yazid A. Al Ohaly, Yi Zhang, Severin Rakic, Mariam Hamza, Christopher H. Herbst, Shenglan Tang

**Affiliations:** 1 Australian Research Council Centre of Excellence in Population Ageing Research (CEPAR), University of New South Wales, Sydney, NSW Australia; 2 School of Risk and Actuarial Studies, University of New South Wales, Sydney, NSW Australia; 3 Global Health Research Center, Duke Kunshan University, Kunshan, Jiangsu China; 4 SingHealth Duke-NUS Global Health Institute, Duke-NUS, Singapore, Singapore; 5 World Bank, Washington, D.C. USA; 6 General Directorate for National Health Economics and Policy, Saudi Health Council, Riyadh, Saudi Arabia; 7 Health Research Unit, Gulf Health Council, Riyadh, Saudi Arabia; 8 Duke Global Health Institute, Duke University, Durham, NC USA

**Keywords:** COVID-19, SARS-CoV-2, Pandemic preparedness, Government response, GCC countries, Vaccine

## Abstract

The COVID-19 pandemic has dramatically threatened the Gulf Cooperation Council (GCC) countries which have a large proportion of foreign workers. The governments of GCC countries have proactively implemented a comprehensive set of policy measures, and up to our knowledge, a systematic analysis of qualitative and quantitative evidence on the government response is still lacking. We summarized the GCC countries’ government response and quantitatively measured that response using four indexes—the Government Response Index, the Stringency Index, the Vaccine Index, and the Initial Response Index, to analyse their response for future pandemic preparedness. Overall, the government response of all the GCC countries to the COVID-19 pandemic has been comprehensive, stringent, and timely. Notably, the GCC countries have implemented comprehensive vaccine policies. In addition, they have worked actively to protect foreign workers to improve their access to health services and secure their essential living conditions, regardless of their immigrant status. All the GCC countries dynamically adjusted their response to the evolving COVID-19 epidemiological burden and started to relax the stringency of the control policies after the Omicron wave, though the governments had different response magnitudes as measured by the four indexes. These findings have provided several important lessons for future pandemic response and preparedness for countries with similar economic, demographic, and health contexts in (1) prompt actions of containment and closure policies with dynamic adjusting, (2) strengthening health system policies, (3) comprehensive vaccination policies with universal access, (4) equitable and free access to testing, diagnosis, and treatment for all, and (5) strengthening the resilience of health systems.

## Background

COVID-19 has infected hundreds of millions worldwide since it was first reported in 2019 and declared as the Public health Emergency of International Concern on Jan 30, 2020 [1]. In approximately three months, the disease spread to 177 countries, with new cases in new countries emerging daily [2]. The first SARS-CoV-2 variant, Alpha, was discovered in South Africa in November 2020. Five major variants of concern (VOC) defined by the World Health Organization (WHO) were identified by the end of May 2021; these included Alpha (B.1.1.7), Beta (B.1.351), Gamma (P.1), Delta (B.1.617.2) and Omicron (B.1.1.529). The Delta and Omicron variants are currently spreading at the time of writing this article [3]. The global confirmed cases and deaths toll had risen to over 521 million and 6.2 million, respectively, by May 20, 2022 [4]. Nevertheless, the death rate has decreased dramatically since the Omicron variant started spreading in late 2021 [5]. In order to contain COVID-19, many governments have actively taken a set of actions—such as testing, tracing high-risk people, isolating patients, quarantining, encouraging social distance, and implementing lockdowns and travel bans [6, 7]. These measures were implemented very strictly at the beginning of the pandemic, and have been adjusted accordingly with changing epidemiological situations. In addition, mass vaccination has been another critical containment strategy after several vaccines became available to the public in early 2021 [8].

The Gulf Cooperation Council (GCC) countries—namely, United Arab Emirates (UAE), Bahrain, Saudi Arabia, Oman, Qatar, and Kuwait—are a unique group of countries located in the Arabian Gulf, characterized by rich natural resources, per capita high-income level, a young population structure, and a significant proportion of foreign workers [9]. Saudi Arabia, among all GCC countries, has the largest population size with 36.4 million people in 2022, followed by UAE with 9.4 million population, Oman 4.6 million, Kuwait 4.3 million, Qatar 2.7 million, and Bahrain 1.5 million. Overall, the median gross domestic product (GDP) per capita in the GCC countries was US$20,260 in 2020 [10]. The percentage of people ages 65 years and older accounted for only between 1 and 3 percent of the GCC total population in 2020 [11]. Another distinctive characteristic of the GCC countries is the high percentage of foreign workers, which, on average, consisted of over 76 percent of the total employed population, and 52% of the total population respectively in the GCC countries in 2020 [12–14]. These workers usually earn lower wages, and have poorer access to health care and less job security than nationals [15, 16].

The pandemic of COVID-19 has posed a significant threat to the GCC countries. The first case of COVID-19 in these countries was detected in the UAE on January 29, 2020; and most other GCC countries began to see instances in late February 2020 [17]. In total, the GCC countries had over 3.57 million cases and 20,000 deaths by March 31, 2022 [4]. Oil prices dropped to an unprecedented low in 2020 when the pandemic started, which was a catastrophic economic blow to all the GCC countries [18]. In addition, studies reported COVID-19’s economic and social impacts on the foreign workers in several GCC countries, where most of them were working in the highly affected industry, e.g. construction, domestic service. Many foreign workers worked in the service sector with very dense living conditions. Such environmental risks made them more vulnerable to COVID-19 infection than nationals [15, 16].

As per our knowledge, systematic analysis of qualitative and quantitative evidence on various dimensions of the government response in the GCC countries is still lacking. With previous experience in combating MERS-CoV in 2012, the governments of the GCC countries acted swiftly at the beginning of the pandemic. They started to take comprehensive control measures in early February 2020 [19]. The confirmed cases and deaths have been well under control, despite the surge of cases caused by the Omicron variant in 2021. Previous studies have qualitatively summarized and analysed the efforts by governments of the GCC countries to flatten the curve [15, 19–23]. Nonetheless, little evidence is available to quantify various dimensions of the government response, and no systematic analysis, that integrates qualitative and quantitative evidence, is available.

This study aims to (1) explore the government response in different GCC countries to the COVID-19 pandemic, including containment measure policies, health system policies, vaccination policies, and economic policies; (2) quantitatively evaluate the government response in the GCC countries using four indexes (the Government Response Index, the Stringency Index, the Vaccine Index, and the Initial Response Index) to assess how GCC countries dynamically adjust their responses according to the emerging SARS-CoV-2 VOCs and evolving COVID-19 epidemiological burden; and (3) based on qualitative and quantitative evidence, distil good practices and experiences and discuss implications for future actions with effective responsiveness and preparedness for the emerging and re-merging epidemic/pandemic.

## Approach

The analysis was conducted among all the six GCC countries with high-quality secondary data. We collected COVID-19 control strategies, policies, and measures taken by the governments from government official websites, peer-reviewed publications, grey literature, and credible news media. The COVID-19 epidemiological data, including new and cumulative cases and deaths, were collected from the WHO Coronavirus (COVID-19) Dashboard [4]. We also calculated the cases and deaths relative to the size of the population, using the projected population size in 2021 from the World Population Prospects 2019 study of the United Nations [24]. The data on indexes to evaluate the government response were collected from the Oxford COVID-19 Government Response Tracker (OxCGRT) at the Blavatnik School of Government, University of Oxford [25]. We restricted the data collection to the duration from January 1, 2020 to March 31, 2022; covering the initial outbreak and the subsequent transmission of SARS-CoV-2 VOCs.

We used four indexes to quantitatively measure the government response in the GCC countries. These four indexes are the Government Response Index, the Stringency Index, the Vaccine Index, and the Initial Response Index. The Government Response Index and Stringency Index were developed by the OxCGRT team based on relevant policy indicators (Tables 1 and 2). All these indicators were binary or on an ordinal scale. A binary flag label system was used to further indicate the scope of the measure. The Government Response Index measures the overall government response by their policies in containment and closure, health systems, and economy. The Stringency Index measures the variation of the strictness of policies enforced with administrative power that mainly restrict people’s mobility, which primarily included containment and control policies. Our study team further developed the Vaccine Index based on the four vaccine policy indicators (Tables 1 and 2) included in the OxCGRT. These four indicators captured vaccine prioritization, availability, financing, and compulsory requirement policies in order to evaluate vaccine-related measures comprehensively. Our study team previously developed the Initial Response Index by incorporating timeliness into the Government Response Index to determine the swiftness of governments’ response early in the pandemic. It used the same indicators as Government Response Index except for indicator H7, as vaccination policies were not available during the initial stage of the pandemic in early 2020. The reasons to use these four indexes were (1) the nature of capturing key aspects for COVID-19 policies, namely, the substantiveness, stringency, timelessness of policies and the use of vaccines as the key tool to end the pandemic, and (2) the good quality and availability of data in the real world in generating these indexes. More information about the methods on OxCGRT indicators, Government Response Index, and Stringency Index, can be found on the OxCGRT website at https://github.com/OxCGRT/covid-policy-tracker/blob/master/documentation/index_methodology.md [26]. The detailed development methodology of the Initial Response Index was published elsewhere [6]. In this study, patients and the public were not involved in the research design nor in the result dissemination process.

**Table 1:** The OxCGRT indicators of the government response index, stringency index, and the vaccine index. *Source*: OxCGRT

Index	Containment and closure policies								Economic policies		Health system policies						Vaccination policies			
	C1	C2	C3	C4	C5	C6	C7	C8	E1	E2	H1	H2	H3	H6	H7	H8	V1	V2A	V3	V4
Government Response Index	X	x	x	x	x	x	x	x	x	x	x	x	x	x	x	x				
Stringency Index	X	x	x	x	x	x	x	x			x									
Vaccine Index																	x	x	x	x
Initial response index	X	x	x	x	x	x	x	x	x	x	x	x	x	x		x

**Table 2:** Summary of government response in the GCC countries. *Source*: OxCGRT and relevant literature

Category	OxCGRT ID	Measures description	Response summary
Containment and closure policies	C1	School closing	All GCC countries closed to school and reopened in November 2021 [21, 36]
	C2	Workplace closing	All GCC countries closed non-essential businesses and reopened while maintaining social-distancing measures after the curve was flattened [21, 28]
	C3	Cancelling public events	All GCC countries banned large public events such as concerts and religious and sporting events during the initial phase of the COVID-19 outbreak. Subsequently, public events were open to those who had completed vaccination [28]
	C4	Restrictions on gatherings	All GCC countries placed restrictions on social gatherings, such as the postponement of large public events and mass gatherings such as weddings, and restrictions on religious mass gatherings, including pilgrimage [19, 28]
	C5	Closing of public transport	Bahrain and the UAE: Public transportation was fully functional with restrictions Kuwait, Oman, Qatar, and Saudi Arabia: Public transportation was initially suspended [28]
	C6	Stay-at-home requirements	The UAE, Saudi Arabia, Oman, and Kuwait: Indefinite curfews were imposed, and major cities were sealed off; national curfews in these four countries lasted from March 2020 until May to August 2021 [21, 28] Bahrain and Qatar: No curfews were imposed [28]
	C7	Restrictions on internal movement	Saudi Arabia: Issued a ban on movement between 13 provinces and, effective on September 2021, only fully vaccinated passengers were allowed to fly on domestic flights The UAE: All passengers entering Abu Dhabi must have a negative COVID-19 PCR test result issued within 48 h Omen: Issued a ban on interstate travel, and only people vaccinated with at least one dose were allowed to enter Musandam Governorate & Governorate of Dhofar (Khareef) Kuwait: Vaccinated people can enter all places, but unvaccinated people can enter only pharmacies, supermarkets, medical centres, and hospitals Bahrain and Qatar: No restrictions found [21]
	C8	International travel controls	All GCC countries (except Bahrain) initially suspended international flights but then gradually resumed, with the timing of resumes varying from country to country [21]
Health system policies	H1	Public information campaigns	All GCC countries had a timely assessment of the risks of COVID-19, and transparent communication messaging was implemented at national and subnational levels. Oman’s response did not include risk communication team/health professionals at the subnational level [19]
	H2	Policy on who has access to testing	PCR tests are widely available for all GCC countries [28]
	H3	Policy on contact tracing after a positive diagnosis	All GCC member states established a daily practice of monitoring COVID-19 cases, mortality, and contact tracing, as well as aggregating weekly and monthly data [19]
	H4	Announced short-term spending on the healthcare system, e.g. hospitals, masks, etc	All six GCC countries strengthened healthcare facilities, designated hospitals for treating COVID-19 patients, enforced infection control procedures and visual triage, and monitored the capacity for isolation beds, equipment, human resources, and critical medical supplies [19]
	H5	Announced public spending on COVID-19 vaccine development	—
	H6	Policies on the use of facial coverings outside the home	All implemented indoor mask-wearing requirements [28]
	H7	Policies for vaccine delivery for different groups	All GCC countries have policies to determine the priority of vaccination among population groups [28]
	H8	Protecting older people (as defined locally) in long-term care facilities and/or the community and home setting	Bahrain, the UAE, and Qatar have recorded policies to protect older people Saudi Arabia, Kuwait, and Oman: No relevant information was found [28]
Vaccination policies	V1	The ranked position for different groups within a country prioritization plan	All the GCC countries have a step-by-step plan to vaccinate residents based on the prioritization plan in the country. Similarly, people most at risk of developing severe symptoms (e.g., senior people, people with chronic diseases) and people most at risk of getting infected (e.g., health care workers) are the top priorities [28]
	V2	Categories of people—regardless of their position in a prioritized rollout plan—are currently receiving vaccines	Nationals and non-nationals in all the GCC countries are receiving vaccines in the GCC countries, despite their position in the prioritization order [28]
	V3	How vaccines are funded for each category of people identified in OxCGRT ID category V2 as currently receiving vaccines	Vaccines are free in the GCC countries for all citizens and residents [28]
	V4	Vaccine requirements for workers	All GCC countries have had a mandatory vaccination requirement for all people involved in indoor activities [28]
Economic policies	E1	Providing direct cash payments to people who lose their jobs or cannot work	Bahrain: Provides financial aid for taxi and bus drivers, kindergarten caregivers, and driving instructors Kuwait: Establishes a mechanism to secure the minimum income that ensures the cost of living for workers affected by the current crisis and linked to contracts Oman: Provides temporary income support to insured Omanis who lost their employment involuntarily and who seek to become re-employed Qatar: All workers in isolation, quarantine, or receiving treatment are paid their basic salary and receive their allowances irrespective of whether they are entitled to sick leave benefits Saudi Arabia: Issued a decision to cover 60% of salaries in the private sector. Wage support is extended only through July 2021 The UAE: Employers who lose their jobs under unforeseen circumstances will reportedly receive 60 percent of their salary, or up to $5,445 (AED 20,000) monthly for a limited time, helping maintain a rolling income [21]
	E2	Freezing financial obligations for households	All the GCC countries have developed related economic policies to relieve the financial burden among households, though the intensity varies [21]
	E3	Announced economic stimulus spending	All GCC countries introduced billion-dollar stimulus packages to help boost economies [21]
	E4	Announced offers of COVID-19-related aid spending to other countries	The GCC countries have come together to support global equitable access to COVID-19 vaccines, with US$221 million in funding pledges and US$50 million in in-kind support [37]

PCR, polymerase chain reaction; —, not available

## Epidemiological burden of COVID-19 in the GCC countries

Since the onset of COVID-19, a total of 3,574,147 cases and 20,296 deaths were reported in the GCC countries by March 31, 2022, with country variations in epidemiological burden. The UAE had the highest absolute total number of cases (890,987), followed by Saudi Arabia, Kuwait, Bahrain, Oman, and Qatar. The total deaths were highest in Saudi Arabia (9,043) and lowest in Qatar (677). Nonetheless, after adjusting for population size, Bahrain had the highest burden as it yielded 316,737 cases and 841 deaths per 100,000 population. Meanwhile, Saudi Arabia had the lowest incidence rate (21,242 per 100,000 population), while the UAE had the lowest death rate (230 per 100,000 population) during the study period.

**Fig. 1:**
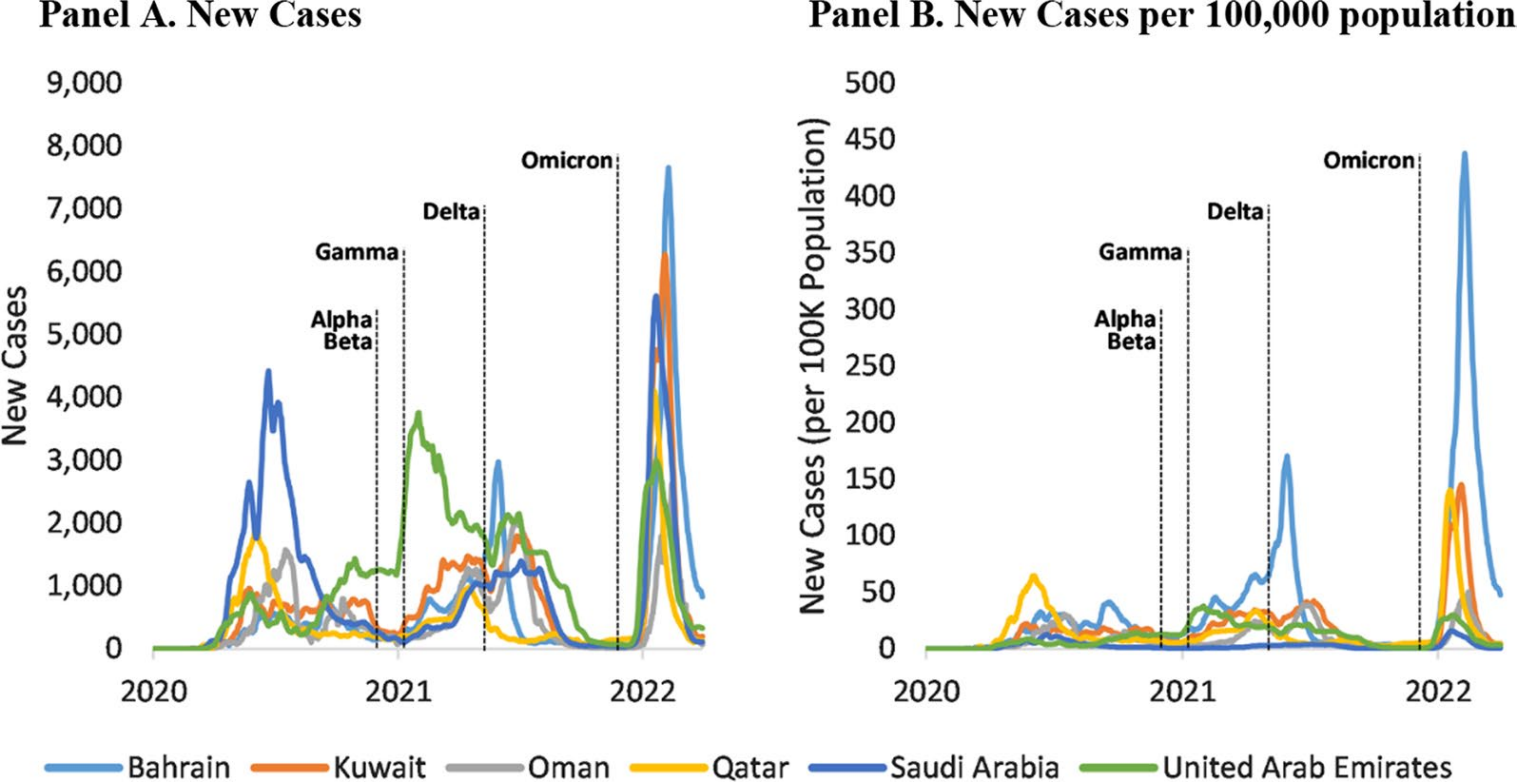
Daily New Cases (7-Day Average) of COVID-19 GCC Countries, from January 2020 to March 2022. *Data source:* WHO Coronavirus (COVID-19) Dashboard

**Fig. 2:**
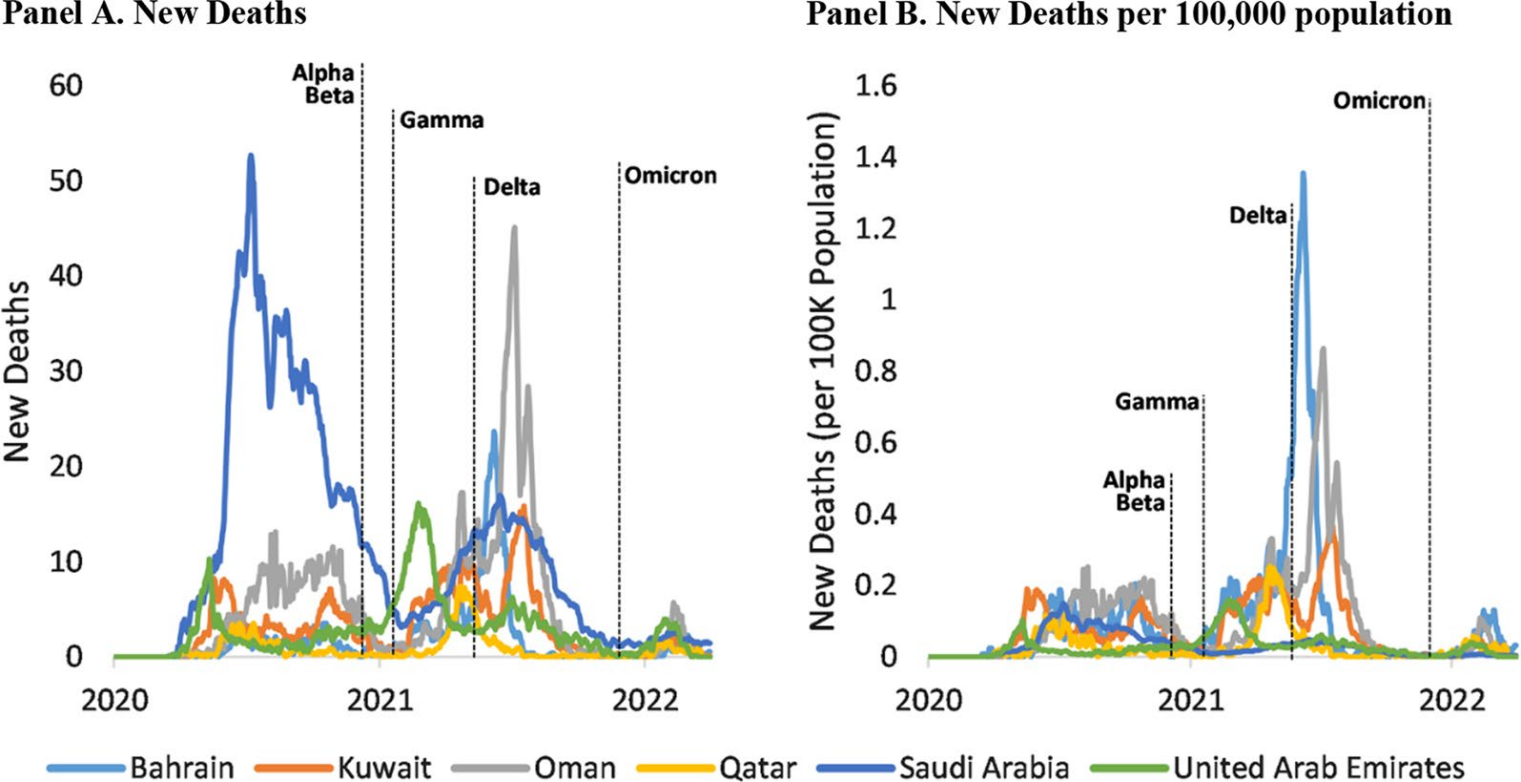
Daily New Deaths (7-Day Average) of COVID-19, GCC Countries, January 2020 to March 2022. *Data source:* WHO Coronavirus (COVID-19) Dashboard

The GCC countries shared a similar pattern in the change of the COVID-19 epidemiological burden with the spread of SARS-CoV-2 variants. There was a surge of new cases in all GCC countries every time transmission of a new variant started (Fig. 1). The Omicron variant has caused most new cases throughout the study period. Similarly, there was a surge of deaths with the spread of each new variant, although this dropped dramatically since the Omicron variant appeared (Fig. 2). Specifically, the daily new death rate was below 3 per 100,000 population for all six countries from January 2020 to mid-May 2021 [27]. Nevertheless, daily deaths quickly increased and peaked between June and July 2021 after Delta was declared a VOC by the WHO. The highest daily death rate was observed in Bahrain (1.66 per 100,000 population) on June 2, 2021. It had remained well under 0.1 per 100,000 population since late November 2021, when the Omicron variant started to spread.

## Qualitative summary of the GCC countries’ government response to COVID-19

All GCC countries’ governments have actively responded to the COVID-19 pandemic by implementing a series of policies (on containment and closure, health system, vaccination, and economic policies) during the study period (Table 2).

Containment and closure policies were implemented in all GCC countries, with various levels of stringency. The containment and closure policies were enforced with administrative power to constrain people’s mobility, such as workplace closure and travel control, for the purpose of reducing transmission among the population. All the GCC countries had implemented the identified eight containment and control policies, despite their stringency differences [28]. For instance, the UAE, Saudi Arabia, Oman, and Kuwait initiated curfews, and major cities were sealed off when the pandemic started. On the contrary, Bahrain and Qatar did not impose any curfews [21].

All key health system policies, except for investment in vaccine development, have been effectively implemented in the GCC countries. The health system policies were primarily public health measures covering testing, contact tracing, vaccine development and delivery, health system strengthening, wearing facial masks, and implementing special measures to protect the elderly. The stringency of implementation also varied [28]. For example, in terms of measures to protect the elderly, Qatar has published and implemented several guidelines on isolation, nutrition, and care, to protect the elderly [29]. Bahrain has launched mobile units providing home vaccinations against COVID-19 for the elderly and people with special needs [30]. In the UAE, Dubai announced measures to protect staff, such as allowing elderly members of the workforce to work remotely [31].

All six GCC countries had rolled out country prioritization plans to vaccinate their populations (both nationals and non-nationals) free of charge after vaccines became available in November 2020. Similarly, people most at risk of developing severe symptoms (for example, seniors, people with chronic diseases, and so on) and people most at risk of infection (for example, healthcare workers) had been the top priorities to be vaccinated [28]. Foreign workers had also been granted free access to vaccines [16]. All GCC countries had a mandatory vaccination requirement for all people involved in daily indoor activities, and some countries targeted broader populations [28]. For example, the UAE required all students over 16 must be vaccinated against COVID-19 to attend school [32]. Saudi Arabia required employees to be vaccinated in order to appear in their workplace; and domestic travellers to be fully vaccinated in order to be able to fly [33, 34]. For the purpose of alleviating the economic burden on enterprises and households, the GCC countries had also offered generous *financial support policies* during the pandemic, including providing direct cash payments, freezing financial obligations, and providing economic stimulus packages [21].

The GCC countries had implemented special policies for foreign workers, who comprise a large proportion of the population and generally have limited access to health services. All necessary measures that cover testing, diagnosis, treatment, and vaccines for COVID-19, including hospitalization, were made accessible to all foreign workers in the six countries regardless of their immigration status [16, 19]. In addition, the GCC countries extended work permits and residency permits for workers who could not return to their home countries because of travel restrictions and provided sheltered places for undocumented workers during the pandemic [21]. For example, in Saudi Arabia, residency permits were extended for three months for expatriates, and the expat levy was exempted [35]. In Bahrain and Kuwait, undocumented workers, were exempted and granted temporary residency [21]. In the UAE and Oman, delay fines and extension of expat manpower’s licenses were waived [21]. In Qatar, the residency permits were renewed automatically [21].

## Quantitative measurement of the GCC countries’ government response to COVID-19

**Fig. 3:**
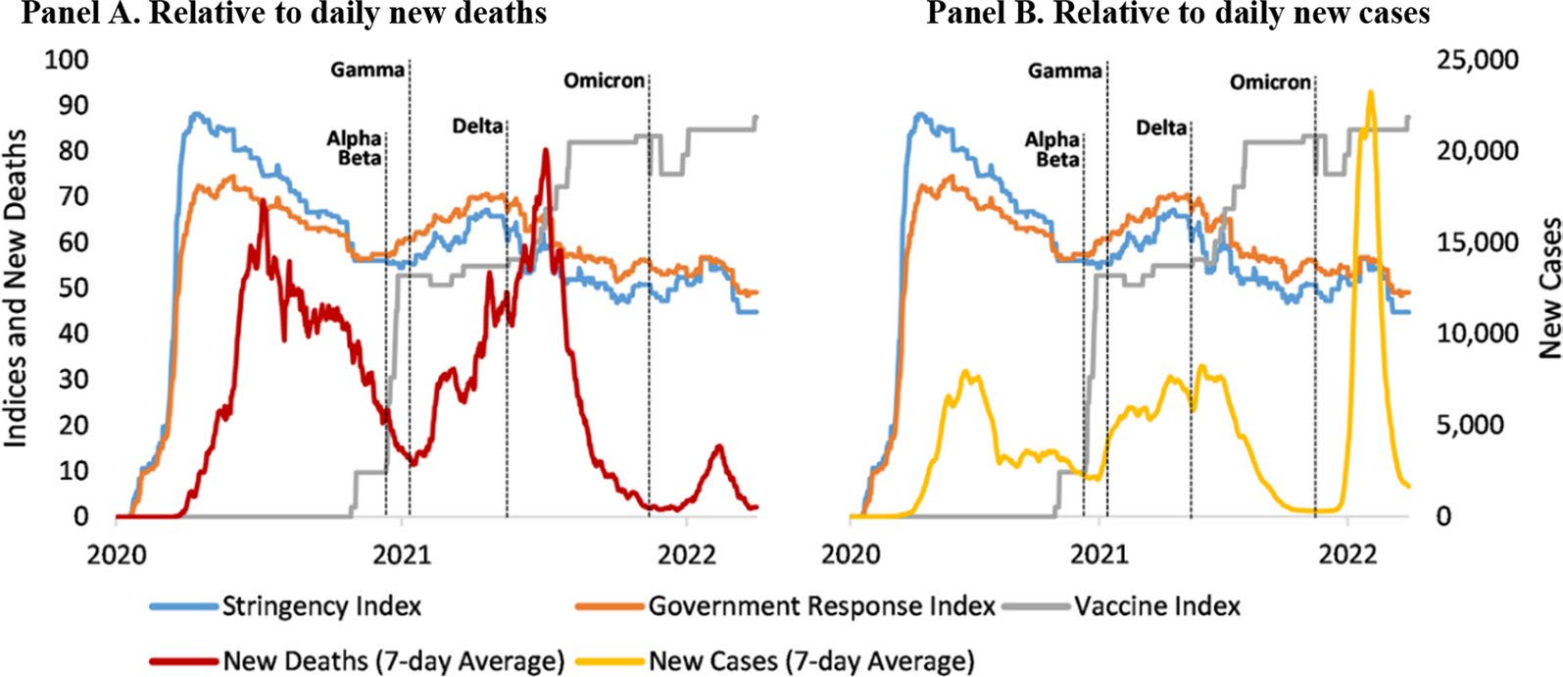
Change in the score of the Aggregated Stringency Index, Government Response Index, and Vaccine Index of the GCC Countries Relative to the COVID-19 Epidemiological Burden, January 2020 to March 2022. *Source:* OxCGRT

**Fig. 4:**
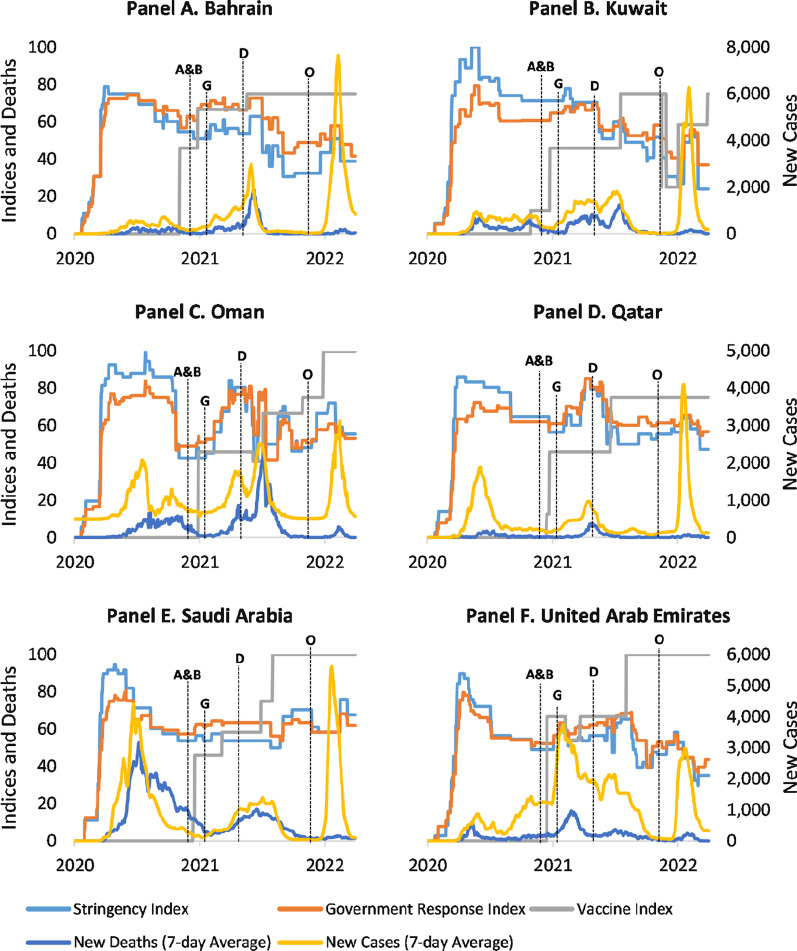
Country Profiles of the GCC Countries in the Change of the Stringency Index, Government Response Index, and Vaccine Index relative to COVID-19 epidemiological burden, Jan 2020 to March 2022. *Source:* OxCGRT. *Note:* VOC (a variant of concern) labels: A&B = Alpha and Beta; G = Gamma; D = Delta; O = Omicron

All the GCC countries had dynamically adjusted their response to the evolving COVID-19 epidemiological burden over the study period (Figs. 3 and 4). We observed a similar trend: the governments had the highest Stringency Index score (close to 90 on a scale of 1 to 100), exhibiting the strictest lockdown-style policies that restrict people's behaviours at the very beginning of the outbreak. The second peak of the Stringency Index score (close to 65) came during the Delta variant period with the surge of new cases. Governments continued to drop in the Stringency Index score—especially after the cases caused by the Delta variant were under control—and did not step up the stringency after the Omicron variant started (scores remained around 50). The Government Response Index, showing how the overall government response varied, followed a variation pattern like that of the Stringency Index. Interestingly, the Government Response Index score was lower than the Stringency Index score at the beginning of the outbreak. Still, it started to surpass the Stringency Index around the end of 2020, when other control measures—especially vaccines—became available. The Vaccine Index score, which shows how vaccine prioritization, availability, financing, and requirements policies varied, increased after the Delta variant appeared and has been kept at an even higher level since the Omicron variant started to dominate. Notably, with the evident increase in the score of the Vaccine Index, the number of new deaths had dropped sharply after the peak caused by the Delta variant.

Though sharing similar response patterns, the GCC countries had different response magnitudes over the study period (Fig. 4). On average, Oman had the highest Stringency Index score (63.1), followed by Qatar, Saudi Arabia, Kuwait, the UAE, and Bahrain throughout the study period. Qatar had the highest Government Response Index score (61.4), followed by Saudi Arabia, Bahrain, Oman, Kuwait, and the UAE. We observed different paces of initiating the government response when the pandemic started, as measured by the Initial Response Index. Oman began to be the fastest among all the GCC countries with an index score of 45.7, followed by Saudi Arabia (38.1), Bahrain (22.2), Qatar (20.6), Kuwait (18.2), and the UAE (13.9). On average, the Vaccine Index score was highest in the UAE (82.5), followed by Saudi Arabia (78.3), Bahrain (69.7), Oman (65.8), Qatar (62.8), and Kuwait (50.4). Notably, the Vaccine Index has remained at 100 in the UAE and Saudi Arabia since October 2021 and in Oman since late December 2021.

## Good practices, lessons, and implications for future preparedness

The government response of the GCC countries to the COVID-19 pandemic has been comprehensive, stringent, and timely. They have all implemented a complete set of policies to respond to the epidemiological and economic consequences of the pandemic. Policies that aim to reduce transmission and mortality include containment and closure policies, health system policies, and vaccination policies. Specifically, the containment and closure policies restrict people’s mobility, while health system policies and vaccination policies aim to use a public health approach to control the pandemic. Economic policies have also been developed to relieve the financial loss to individuals, households, and enterprises caused by the pandemic. The stringency of the response has been kept high, especially at the beginning of the outbreak. All the governments began to act around late January 2020, when there were fewer than 100 local cases per GCC country. To ensure effectively-coordinated efforts in the future to tackle epidemics and strengthen public health efforts, the GCC countries established the new Gulf CDC in Riyadh, Saudi Arabia, in January 2021. The mission of the Center is envisioned to foster harmonization, build knowledge, and generate evidence to enable the prevention of communicable and non-communicable diseases, mitigation of public health emergencies, and promotion of healthy communities across the GCC region [38].

The GCC countries have adjusted the government response dynamically based on the epidemiological burden, the virological characteristics of the SARS-CoV-2 variants, and the availability of vaccines. Similarly, these governments kept the stringency of lockdown-style policies at a very high level, especially when the pandemic started. However, they began to relax these policies when cases went down, especially after the vaccines became available in late 2020. When the Gamma and Delta variants began to circulate, the GCC countries intensified their health systems and vaccination policies while keeping the stringency of the containment and closure policies at a moderately high level. After the Omicron variant started to hit the GCC countries, the governments stepped up their vaccination policies as their primary response while relaxing other policies to restore the economy. Boosting economics became the top priority after the pandemic death rates were well under control.

Large proportion of foreign workers was a challenge for COVID-19 control in GCC countries, and governments have developed and implemented tailored policies to address this. Compared with nationals, foreign workers usually have lower socioeconomic status, worse living and health conditions, and are prone to SARS-CoV-2 infections [16]. Singapore has the same demographic situation and the uncontrolled spread of SARS-CoV-2 among foreign workers, who lived in high-density accommodations, contributed to the surge of cases in April and May 2020 [39]. The governments of GCC countries have learned this lesson and worked actively to formulate and implement effective policies for the foreign workers to improve their access to health services and secure their essential living conditions, regardless of their immigrant status. This aligned with the recommendations put forth by the International Labour Organization and World Health Organization during the early phase of the pandemic [40, 41]. The governments have provided free testing, diagnosis, and treatment for these workers. Furthermore, they extended work and residency permits for workers who could not return to their home countries due to travel controls. In addition, some governments also provided sheltered places for undocumented workers during the pandemic.

The GCC countries have proactively and effectively implemented comprehensive vaccination policies targeting vulnerable and high-risk populations. The vaccination policies conducted in these countries primarily cover (1) developing a country prioritization plan to deliver the vaccines to populations with different risks of infection and developing severe symptoms; (2) ensuring the availability of vaccines among the population regardless of their prioritization order; (3) provision of sufficient funding for each recipient category in the prioritization plan to be freely vaccinated; (4) stating compulsory requirements to facilitate vaccination, such as restricting indoor activities and travel among the unvaccinated. Prominently, these countries have kept the vaccination campaign momentum after the surge of cases caused by the Omicron variant, especially the UAE, Saudi Arabia, and Oman. Notably, the share of people who received at least two doses of vaccines had reached 96.2 percent in the UAE by the end of March 2022, which was the highest rate worldwide [42]. Globally, Singapore and Portugal were the second and third most vaccinated countries, at 90.3 percent and 86.8 percent; respectively, and during the same period [42].

Several important causes shape the effective government response of the GCC countries. First, previous experience in dealing with the MERS-CoV had helped the region to strengthen its preparedness and response efforts, facilitating a faster and more effective response against the COVID-19 pandemic when it was initiated [18]. Measures such as lockdowns of major cities, school closures, and suspension of flights were implemented quickly, as those Asian countries or regions—such as China (Mainland, Hong Kong and Taiwan) and Singapore, which had previous experience in controlling SARS—did [6, 43–45]. Second, the region has used its available financial resources to develop a set of socioeconomic policies and tools for effective responses. Sufficient funding has been used to support the implementation of all the control and economic policies, which is especially important regarding securing vaccines and improving coverage. A recent study further demonstrates that higher GDP per capita can significantly predict lower cumulative rates of SARS-CoV-2 infection, based on data from 177 countries [46]. In addition, the carefully-designed and comprehensive vaccination policies and robust implementation, as mentioned above, are other critical factors in reducing death rates.

The study findings have provided several important lessons for other pandemic responses and preparedness, not only for GCC countries but also for other countries with similar economic, demographic, and health system contexts. First, it is essential to promptly activate and implement containment and closure policies under the government’s strong leadership to restrict people’s mobility at the very beginning of the pandemic or epidemic. It is essential to reduce interpersonal transmission when there is limited knowledge about a new virus. Second, the stringency of the containment and closure policies needs to be dynamically adjusted based on the epidemiological burden, the virological features of different virus variants, and the availability of vaccines and medicines. Strengthening health system policies, such as testing and contact tracing, is the key to maintaining the gains achieved when it is time to relax the stringency of lockdown-style policies and to prepare for opening up. Third, it is essential to proactively implement comprehensive vaccination policies to improve coverage when vaccines are available and provide sufficient funding to ensure free access to vaccines. Fourth, it is crucial to ensure equitable and free access to testing, diagnosis, and treatment for all residents, regardless of their nationality and immigration status. Particular attention should be paid to vulnerable or marginalized populations, such as migrant workers and others with lower socioeconomic status living in poor and high-density accommodations. Fifth, strengthening the resilience of health systems—especially in governance, financing, human resources, and service delivery [47]—should be integrated as part of routine work to enhance pandemic response capacity. The four elements of resilience in highly effective country responses to COVID-19, as outlined in a recent study among 28 countries, are (1) activating comprehensive responses, (2) adapting health system capacity, (3) preserving health system functions and resources, and (4) reducing vulnerability [48]. Countries can use these elements to guide and monitor the system resilience strengthening throughout the process.

## Conclusions

The GCC countries have effectively implemented a set of containment and closure, health systems, vaccination, and economic policies in response to the COVID-19 pandemic. However, some disparities among countries have been observed in terms of response magnitude. The past often informs the future. The GCC countries have learned from their past experiences in epidemic/pandemic control, which helped them to tackle the challenges of the COVID-19 pandemic over the past two years. Countries worldwide may learn from GCC countries in one way or another in their future pandemic preparedness, as summarized here, and integrate the process of strengthening the resilience of the health systems into daily work to enhance pandemic response capacity.

## Data Availability

The datasets analysed during the current study are available at the OxCGRT website, https://covidtracker.bsg.ox.ac.uk/.

## Abbreviations

GCC: Cooperation Council for the Arab States of the Gulf, or Gulf Cooperation Council
GDP: Gross Domestic Product
GHC: Gulf Health Council
KSA: Kingdom of Saudi Arabia
MERS-CoV: Middle East Respiratory Syndrome Coronavirus
OxCGRT: Oxford Covid-19 Government Response Tracker
PCR: Polymerase Chain Reaction
SHC: Saudi Health Council
UAE: United Arab Emirates
VOCs: Variants of Concern
WB: World Bank
WHO: World Health Organization
